# Influenza vaccines licensed in the United States in healthy children: a systematic review and network meta-analysis (Protocol)

**DOI:** 10.1186/2046-4053-1-65

**Published:** 2012-12-29

**Authors:** Gabriela J Prutsky, Juan Pablo Domecq, Tarig Elraiyah, Zhen Wang, Lisa A Grohskopf, Larry J Prokop, Victor M Montori, Mohammad Hassan Murad

**Affiliations:** 1 Knowledge and Evaluation Research Unit (KER), Mayo Clinic, 200 First Street SW, Rochester, MN, 55905, USA; 2 Unidad de Conocimiento y Evidencia (CONEVID), Universidad Peruana Cayetano Heredia, 430 Honorio Delgado Ave, San Martin de Porres, Lima 31, Peru; 3 Division of Preventive, Occupational and Aerospace Medicine, Mayo Clinic, 200 First Street SW, Rochester, MN, 55905, USA; 4 Division of Endocrinology, Diabetes, Metabolism, Nutrition, Mayo Clinic, 200 First Street SW, Rochester, MN, 55905, USA; 5 Mayo Clinic Libraries, Mayo Clinic, 200 First Street SW, Rochester, MN, 55905, USA; 6 Influenza Division, Centers for Disease Control and Prevention (CDC), 1600 Clifton Road, Atlanta, GA, 30333, USA

**Keywords:** Influenza, Influenza vaccine, Children, Systematic review

## Abstract

**Background:**

Influenza is an acute respiratory illness caused by influenza viruses, which occurs in epidemics worldwide every year. Children are an important target for prevention methods, including vaccination. While evidence about the decision on whether to vaccinate healthy children is robust, evidence supporting the decision of which of available vaccines to use remains unclear.

This review will summarize the evidence about the efficacy and safety of the available vaccines for seasonal influenza licensed in the United States for use in healthy children.

**Methods/design:**

An umbrella systematic review (SR) and network meta-analysis will be conducted of randomized controlled trials (RCTs). We will search for SRs to identify parallel RCTs evaluating inactive and/or live attenuated influenza vaccines licensed in the United States for use in healthy children to prevent influenza. Subsequently, we will update the literature search of the selected SRs to the present time to capture recent controlled studies. To complement the work focused on harms, we will also select observational studies focusing on post marketing retrospective studies. Inclusion will not be limited by language, publication date or publication status. To identify additional candidate studies, we will review the reference lists of the eligible primary studies and narrative reviews; we will query the expert members of the Advisory Committee on Immunization Practices and review references from their previous statement. Additionally, we will review the reports from the Institute of Medicine on the adverse effects of vaccines. Two reviewers will independently determine study eligibility and will extract descriptive, methodological (using the Cochrane risk of bias tool for RCTs and the Newcastle–Ottawa scale for observational studies) and efficacy data. When possible, we will conduct meta-analyses and network meta-analyses by combining indirect and direct comparisons.

We will evaluate heterogeneity using the I^2^ statistic and the agreement of indirect comparisons and direct evidence. We will report the Cochrane Q test to determine the statistical significance of heterogeneity.

The overall quality of evidence will be assessed following the GRADE (Grading of Recommendation, Assessment, Development and Evaluation) approach.

**Discussion:**

Our systematic review will allow patients, clinicians, guideline developers and policy makers to make evidence-based choices between the two available vaccine options, by providing information regarding benefits and harms of these types of vaccines.

## Background

Influenza is an acute respiratory illness caused by influenza A or B viruses, which occurs in seasonal outbreaks worldwide each year, causing substantial morbidity and mortality—between 3,000 and 49,000 deaths [1] and over 200,000 hospitalizations [2] annually. While the majority of influenza-related deaths occur among adults aged 65 years and older [1], children are also at risk of severe disease, particularly those under 5 years of age [3].

Children are the major source for the diffusion of influenza viruses in the community [4,5]. Some studies showed socioeconomic benefits from influenza vaccination in healthy children, both for them and their adult household contacts [6]. For these reasons, much emphasis has been placed on preventing influenza in children. The adoption of universal annual influenza vaccination of children could reduce the burden of influenza illness among vaccinated children, unvaccinated household and school contacts, and reduce community transmission, thereby reducing severe illness among high risk adults [7].

Currently available seasonal influenza vaccines may be divided into two groups:

•Inactivated influenza vaccines (IIV): These include subunit vaccines (containing only surface antigens); split-virion vaccines (in which virus is inactivated with disrupting agents, yielding surface and internal antigens); and whole virion inactivated vaccines (containing whole inactivated viruses). Among these, only split-virion vaccines are currently licensed in the United States. Most are administered intramuscularly; one intradermal preparation is available. IIV is available from a number of different manufacturers; age indications for the different preparations differ. IIV preparations are available for children as young as six months of age.

•Live-attenuated (cold-adapted) influenza vaccines (LAIV): These intranasally-administered vaccines contain virus which can multiply only in the nasal passages. LAIV is licensed for persons 2 through 49 years old and is recommended for healthy, non-pregnant individuals who do not have chronic medical conditions, due to relative lack of data in the other populations [8].

The Centers for Disease Control and Prevention (CDC) Advisory Committee on Immunization Practices (ACIP) [8] and the American Academy of Pediatrics (AAP) [9] recommend annual influenza vaccination for all children aged ≥6 months. While the evidence supporting the benefits of vaccinating healthy children to prevent influenza is robust, the evidence to support the decision on which vaccine form to use with whom remains unclear; ACIP currently expresses no preference for one vaccine over the other [5].

### Study objectives

This systematic review (SR) aims to summarize the evidence concerning the efficacy and safety of US-licensed seasonal influenza vaccines for healthy children 6-months through 18-years old. Evidence for the comparative benefits of LAIV and IIV among children 2- through 18-years old will also be examined.

This evidence will help guideline developers, clinicians and patients in choosing the most suitable vaccine for each age group based on tradeoffs between harms and benefits.

## Methods/design

Considering the availability of multiple well-conducted SRs addressing this question, we will start by conducting an umbrella systematic review [10,11] to identify existing SRs and select the most recent and comprehensive ones. We will evaluate each systematic review using the Assessment of Multiple Systematic Reviews (AMSTAR) criteria [12] particularly emphasizing the quality and comprehensiveness of the search strategy. We will also consider their inclusion and exclusion criteria. Subsequently, we will update and, if needed, modify the literature search of the selected SRs to the present time to capture recent controlled studies (randomized controlled trials (RCTs) and non-randomized studies). We will search electronic databases (Ovid Medline, OVID EMBASE, OVID Cochrane Library, Web of Science, Scopus and PsycInfo) from the last search date stated in the included SRs through the present time. With input from study investigators with expertise in conducting SRs (MHM, VMM), a reference librarian (LP) and the first author (GP) will design and execute these electronic search strategies using controlled vocabulary and text words. To identify additional candidate studies, we will review the reference lists of the eligible primary studies, narrative reviews and systematic reviews; and we will query the expert members of the ACIP Influenza Work Group and review the references from the previous ACIP Influenza Statement. Additionally, we will review the Institute of Medicine (IOM) report on vaccine adverse effects [13-15] to capture other possible harm evidence. The two search strategies (for SRs and individual studies) are given in the Additional file 1.

### Eligibility criteria

We will include parallel-design RCTs that compared LAIV and IIV to each other or to placebo. Eligible trials must evaluate the efficacy of seasonal influenza vaccines (not pandemic vaccines) licensed in the United States and administered as recommended by the ACIP, and must enroll healthy children (<18 years). We will exclude vaccinations not administered according to ACIP recommendations (for example, LAIV given to children under 2 years of age). Evidence concerning adjuvanted vaccines, subunit vaccines, and whole-virus vaccines will be excluded.

For evidence of harm, we will select observational studies focusing on post marketing retrospective studies. These studies typically have a larger sample size which is essential to detect rare adverse events caused by the vaccine, such as Guillain-Barre syndrome or anaphylaxis, with incidence less than 10 cases per million [16].

### Study selection

**Figure 1:**
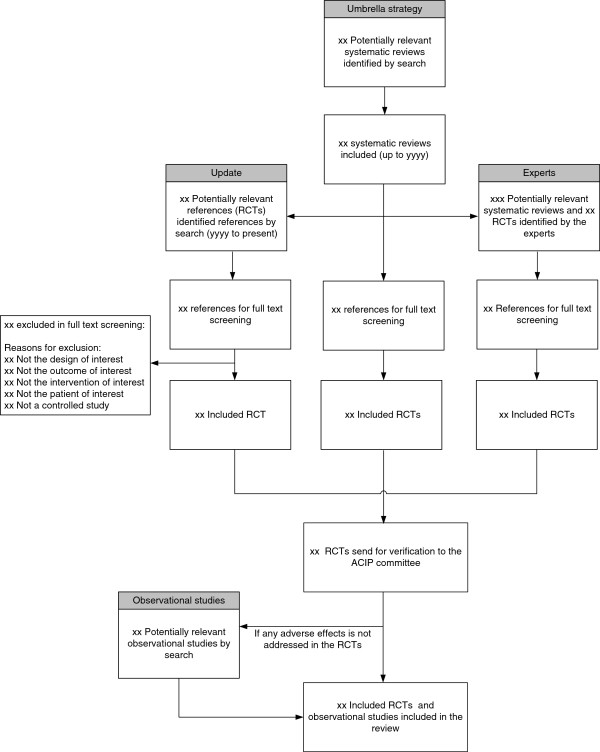
Summary of the study selection process.

Search output will be uploaded into an online reference management system (DistillerSR, Ottawa, Canada) to allow fast and transparent processing with better tracking and real-time evaluation of inter-reviewer agreement and progress of reviewers. Two reviewers working independently will consider the potential eligibility of each of the abstracts and titles that will result from executing the search strategy. Reviewers will request the full text versions of all potentially eligible studies. The full text of papers on which there is disagreement will also be retrieved for evaluation. Two reviewers working independently will consider the full text reports (all available versions of each study) for eligibility. The reviewers will calibrate their judgments using a smaller set of reports. Subsequently, disagreements will be resolved by consensus; if not possible, by arbitration by a third reviewer. Agreement will be measured using the kappa or phi statistic, as appropriate (the latter is appropriate when the distribution of the feature under evaluation is extremely common or rare). This process is represented in Figure 1.

### Data collection and extraction

We will collect data in predefined electronic forms designed using the online reference management system. Two reviewers separately and independently will extract full descriptions of participants enrolled, the interventions they received (vaccine description, doses, route), and the measure of outcome.

### Outcomes of interest

1. Effectiveness outcomes measures: **a.** Laboratory-confirmed influenza infection, confirmed by polymerase chain reaction or viral culture; **b.** Influenza-like illness (ILI) ; **c.** Medically attended respiratory illness (MARI); **d.** Hospitalization; **e.** Mortality; **f**. Medically attended wheezing.

2. Safety outcome measures: **a.** Fever due to vaccination; **b.** Febrile seizures; **c.** Immediate hypersensitivity/anaphylaxis; **d.** Guillain-Barré syndrome; **e.** Serious adverse effects.

### Quality assessment

To assess the methodological quality of RCTs we will use the Cochrane risk-of-bias tool assessment [17] to determine: how the randomization sequence was generated; how allocation was concealed; whether there were important imbalances at baseline; which groups were blinded (patients, care givers, data collectors, outcome assessors, data analysts); loss to follow-up; whether participants were analyzed as randomized; and how missing outcome data were addressed. We will also evaluate the adequacy of the outcome measurement process. For observational studies the quality will be assessed using the Newcastle–Ottawa scale. No scoring system will be derived.

### Statistical analysis

Relative comparisons will be quantified by means of the relative risk (RR) of outcomes with one vaccine form versus the other. We choose to report relative risk instead of odds ratio since RR is more intuitive to clinicians and patients [1-18]. To pool head-to-head comparisons, we will conduct random effects models using the DerSimonian & Laird method and report point estimates and 95% confidence interval [19]. We will evaluate heterogeneity using the I^2^ statistic, a measure of the total variations of study effect sizes due to true heterogeneity between studies. Considering that thresholds for the interpretation of I^2^ can be misleading we will analyze its importance based on magnitude and direction of effects and strength of evidence for heterogeneity (for example, *P* value from the Cochran’s Q test and confidence intervals for I^2^) [20]. We will report the Cochrane Q test to determine the statistical significance of heterogeneity.

**Figure 2:**
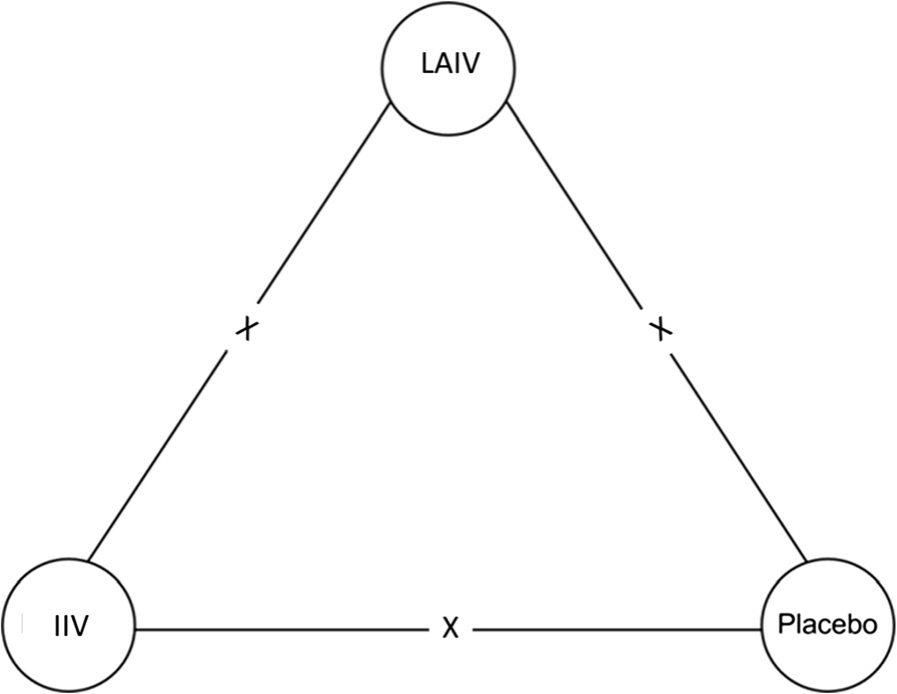
Network of comparisons, graphic representation.

When possible, we will conduct network meta-analyses by combining indirect and direct evidence. The network of comparisons is presented graphically in Figure 2. Due to the small number of comparisons in this study, we are quite confident that the network will have at least one pair of treatments being compared both directly and indirectly. Thus, Lumley’s generalized linear mixed models will be used [21]. The agreement of indirect comparisons and direct evidence, that is, the degree of coherence of the network, will be measured and incorporated in the calculation of the confidence interval of the pooled estimate. If the incoherence is large and network meta-analysis is deemed inappropriate, only estimates from direct evidence will be pooled and reported. In rare cases in which the network of comparisons does not have at least one pair of treatments being compared both directly and indirectly, we will use adjusted indirect comparison (AIC) models instead of generalized linear mixed models [22].

For studies with loss to follow-up, we will apply the intention-to-treat principle and analyze the initial number of patients in the group to which they were randomized. The decision is to preserve randomization benefits in balancing prognosis of trial arms even if it leads to underestimated effect sizes [23].

Statistical analyses will be conducted using STATA version 12.0 (StataCorp, College Station, TX, USA) and R version 2.15.0 (R Foundation for Statistical Computing, Vienna, Austria).

### Subgroup analysis

If sufficient data were available to explore causes of inconsistency and subgroup-treatment interactions, we will construct the following subgroup analyses defined by: **a**. Method used for the diagnosis of laboratory confirmed influenza (PCR versus viral culture versus other); and **b.** Age groups (6 to 23 months, 2 to 8 years, 9 to 18 years).

### Publication bias

Publication bias will be assessed using the Peters linear regression of intervention effects on inverse of study sample size and visual inspection of funnel plots [24].

### Author contact

Data extractors will record the author’s contact information (name and email address) from RCTs and observational studies that fulfilled all the eligibility criteria. We will contact the authors to obtain sufficient details for quality assessment or missing data. If we do not receive the information from the authors we will try to obtain additional information about the methodological characteristics of each study by getting access to each study’s protocol. For industry-sponsored trials we will seek additional information from the current license holder. For studies sponsored by the National Institute of Health we will ask them for the protocol.

### Reporting

This study will be reported in accordance with the recommendations set forth by the Preferred Reporting Items for Systematic Reviews and Meta-Analyses (PRISMA) workgroup [25] and the recommendations developed by the International Society for Pharmacoeconomics and Outcomes Research (ISPOR) [26]. We will present evidence tables for each trial including description of the population characteristics, interventions, methodological quality and main findings.

### Evaluating the quality of evidence

The quality of evidence will be assessed following the GRADE approach. Evidence profiles will be developed using the software GRADE profiler (GRADEpro) as shown in Table 1. This evaluation will include factors of methodological limitations of the studies, such as: overall quality assessment of the included studies, imprecision, indirectness, inconsistency, reporting and publication biases. This will show our confidence in the pooled estimate according to the quality and will be classified as follow: high, moderate, low or very low [27]. Evidence from observational studies could be upgraded if studies showed a large effect size, dose response effect or if the plausible residual confounding is considered to strengthen the association [28]. Unique to judging the quality of evidence in vaccination studies, we will consider additional factors suggested by the Strategic Group of Advisory Experts (SAGE) committee of the World Health Organization which are upgrading the evidence for a population effect or evidence of herd immunity [29].

**Table 1:** Evidence profile

**Quality assessment**							**Number of patients**		**Effect**		**Quality**	**Importance**
**Number of studies**	**Design**	**Risk of bias**	**Inconsistency**	**Indirectness**	**Imprecision**	**Other considerations**	**LAIV**	**IIV**	**RR (95% CI)**	**Absolute**		
**Laboratory confirmed influenza (follow-up; assessed with: PCR or viral culture)**												
	Randomized trials										⊕ ⊕ ⊕ ⊕ HIGH	CRITICAL
**Influenza like illness (follow-up; assessed with)**												
	Randomized trials										⊕ ⊕ ⊕ ⊕ HIGH	IMPORTANT
**Hospitalization (follow-up; assessed with)**												
	Randomized trials										⊕ ⊕ ⊕ ⊕ HIGH	CRITICAL
**Medically attended respiratory illness (follow-up; assessed with)**												
	Randomized trials										⊕ ⊕ ⊕ ⊕ HIGH	CRITICAL
**Fever due to vaccination (follow-up mean, assessed with)**												
	Randomized trials										⊕ ⊕ ⊕ ⊕ HIGH	IMPORTANT
**Guillain-Barre syndrome (follow-up mean, assessed with)**												
	Observational study										⊕ ⊕ ΟΟ LOW	IMPORTANT
**Anaphylaxis (follow-up mean, assessed with)**												
	Observational study										⊕ ⊕ ΟΟ LOW	CRITICAL

**Question:** Should LAIV versus IIV be used for influenza?

**Settings:** healthy children.

CI, confidence interval; IIV, inactivated influenza vaccines; LAIV, live-attenuated influenza vaccines; RR; relative risk.

## Discussion

This network meta-analysis aims at synthesizing the available direct and indirect evidence on the effectiveness and safety of the different vaccines for seasonal influenza in healthy children currently available in the United States. This meta-analysis will allow patients, physician, guideline developers and policy makers to make evidence based choices between the two available vaccine options.

### Limitations and strengths of this study

We will depend on existing SRs to identify individual studies published prior to the date of our search update. Therefore, the comprehensiveness of this SR will depend in part on that of the published SRs. The existing reviews we identified through a preliminary search of the literature seem quite comprehensive (adequate according to the AMSTAR assessment [10]) and include some published by the Cochrane Collaboration [30]. However, we are also supplementing the search strategy by querying experts and reviewing previous ACIP statements and IOM reports to reduce the risk of missing relevant studies.

It is plausible that the variation of influenza strain and virulence from year to year or the matching between the vaccine and the circulating strain will vary across studies. Therefore, the network method of pooling direct and indirect estimates may be associated with a higher level of inconsistency/heterogeneity. In this case, we will abandon the network meta-analysis approach and only use head-to-head RCTs pooled in a traditional random effects model meta-analysis.

These criteria were adopted to provide direct evidence for the comparative effectiveness of contemporary vaccines administered in the United States. The evidence will be less directly applicable to other geographic locations or settings in which the vaccines are administered in a way inconsistent with the ACIP statement.

#### Systematic review status

The systematic review is currently in the data extraction and preliminary analysis phase. We expect completion by October 2012.

## Abbreviations

ACIP: Advisory Committee on Immunization Practices; AMSTAR: Assessment of Multiple Systematic Reviews; GRADE: Grading of Recommendations Assessment, Development and Evaluation; IIV: inactivated influenza vaccine; LAIV: live attenuated influenza vaccine; RCTs: randomized controlled trials; RR: relative risk; SRs: systematic reviews.

## Competing interests

The authors declare they have no competing interests.

## Authors’ contributions

All listed authors contributed substantially to the design of this protocol. All authors read and approved the final manuscript.

## Funding

This study was supported by the Center for Disease Control and Prevention (CDC).

## Supplementary Material

Additional file 1 Search strategies for systematic reviews and individual studies. Click here for file
