# Supplementary material for: Influenza vaccines licensed in the United States in healthy children: a systematic review and network meta-analysis (Protocol)
Source: Syst Rev. 2012 Dec 29;1:65. doi: 10.1186/2046-4053-1-65 (PMC3537683; doi:10.1186/2046-4053-1-65)
Supplement: Additional file 1 — Search strategies for systematic reviews and individual studies. [file 2046-4053-1-65-S1.docx]

Additional File 1

Search strategy for systematic reviews

Ovid

Database(s): Embase 1988 to 2012 Week 01, Ovid MEDLINE(R) In-Process & Other Non-Indexed Citations and Ovid MEDLINE(R) 1946 to Present, EBM Reviews - Cochrane Central Register of Controlled Trials 4th Quarter 2011, EBM Reviews - Cochrane Database of Systematic Reviews 2005 to December 2011Search Strategy:

| **#** | **Searches** | **Results** |
| --- | --- | --- |
| 1 | exp Influenza, Human/dh, dt, pc, su, th [Diet Therapy, Drug Therapy, Prevention & Control, Surgery, Therapy] | 13,767 |
| 2 | exp influenza/dm, dt, pc, rt, su, th [Disease Management, Drug Therapy, Prevention, Radiotherapy, Surgery, Therapy] | 19,311 |
| 3 | (influenza* or flu or flue or grippe or (bronchitis adj epidemic*)).ab,ti,sh,hw,kw. | 186,332 |
| 4 | (therap* or treat* or radiotherap* or surger* or management or prevent*).ab,ti,sh,hw,kw. | 11,955,976 |
| 5 | 3 and 4 | 68,990 |
| 6 | 1 or 2 or 5 | 86,628 |
| 7 | exp "systematic review"/ | 46,439 |
| 8 | (systematic* adj2 review*).mp. [mp=ti, ab, sh, hw, tn, ot, dm, mf, dv, kw, nm, ps, rs, ui, tx, ct] | 109,806 |
| 9 | 6 and (7 or 8) | 1,010 |
| 10 | from 5 keep 68953-68990 | 38 |
| 11 | 9 or 10 | 1,015 |
| 12 | remove duplicates from 11 | 881 |
| 13 | limit 12 to human [Limit not valid in CCTR,CDSR; records were retained] | 851 |
| 14 | limit 13 to yr="2007 -Current" | 500 |
| 15 | limit 14 to humans [Limit not valid in CCTR,CDSR; records were retained] | 500 |
| 16 | limit 15 to (book or book series or editorial or erratum or letter or note or addresses or autobiography or bibliography or biography or comment or dictionary or directory or interactive tutorial or interview or lectures or legal cases or legislation or news or newspaper article or overall or patient education handout or periodical index or portraits or published erratum or video-audio media or webcasts) (Limit not valid in Embase,Ovid MEDLINE(R),Ovid MEDLINE(R) In-Process,CCTR,CDSR; records were retained) | 47 |
| 17 | from 16 keep 1-15 | 15 |
| 18 | 15 not 17 | 485 |

Scopus

| 1 | TITLE-ABS-KEY(influenza* or flu or flue or grippe or (bronchitis W/1 epidemic*)) |
| --- | --- |
| 2 | TITLE-ABS-KEY(therap* or treat* or radiotherap* or surger* or management or prevent*) |
| 3 | TITLE-ABS-KEY(systematic* W/2 review*) |
| 4 | 1 and 2 and 3 |
| 5 | PUBYEAR > 2006 |
| 6 | 4 and 5 |
| 7 | PMID(0*) OR PMID(1*) OR PMID(2*) OR PMID(3*) OR PMID(4*) OR PMID(5*) OR PMID(6*) OR PMID(7*) OR PMID(8*) OR PMID(9*) |
| 8 | 6 and not 7 |
| 9 | DOCTYPE(le) OR DOCTYPE(ed) OR DOCTYPE(bk) OR DOCTYPE(er) OR DOCTYPE(no) OR DOCTYPE(sh) |
| 10 | #8 and not #9 |

Search strategy for individual studies

Ovid

Database(s): Embase 1988 to 2012 Week 07, Ovid MEDLINE(R) In-Process & Other Non-Indexed Citations and Ovid MEDLINE(R) 1946 to Present, EBM Reviews - Cochrane Central Register of Controlled Trials February 2012, EBM Reviews - Cochrane Database of Systematic Reviews 2005 to January 2012Search Strategy:

| **#** | **Searches** | **Results** |
| --- | --- | --- |
| 1 | exp Influenza Vaccines/ | 32,562 |
| 2 | exp influenza/ | 69,528 |
| 3 | exp vaccine/ | 164,276 |
| 4 | exp Vaccines/ | 322,896 |
| 5 | 2 and (3 or 4) | 23,374 |
| 6 | (influenza* or flu or flue or grippe or (bronchitis adj epidemic*)).ab,ti,sh,hw,kw. | 188,722 |
| 7 | (vaccin* or immuni* or innoculat*).ab,ti,sh,hw,kw. | 866,559 |
| 8 | ((influenza* or flu or flue or grippe or (bronchitis adj epidemic*)) adj2 (vaccin* or immuni* or innoculat*)).ab,ti,sh,hw,kw. | 45,576 |
| 9 | 1 or 5 or 8 | 46,372 |
| 10 | exp Vaccines, Attenuated/ | 15,751 |
| 11 | exp Vaccines, Inactivated/ | 9,288 |
| 12 | exp live vaccine/ | 7,111 |
| 13 | exp inactivated vaccine/ | 9,288 |
| 14 | exp placebo/ | 159,343 |
| 15 | exp Placebos/ | 210,207 |
| 16 | placebo*.mp. | 508,565 |
| 17 | 10 or 12 or (live or attenuated or active).mp. | 1,644,803 |
| 18 | 11 or 13 or (killed or inactiv*).mp. | 516,221 |
| 19 | 14 or 15 or 16 | 508,565 |
| 20 | 17 and (18 or 19) | 169,781 |
| 21 | 18 and 19 | 5,499 |
| 22 | 20 or 21 | 173,453 |
| 23 | 9 and 22 | 2,801 |
| 24 | exp controlled study/ | 3,742,834 |
| 25 | exp evidence based medicine/ | 537,849 |
| 26 | evidence-based.mp. | 181,859 |
| 27 | ((control$ or randomized) adj2 (study or studies or trial or trials)).mp. [mp=ti, ab, sh, hw, tn, ot, dm, mf, dv, kw, nm, ps, rs, ui, tx, ct] | 4,783,491 |
| 28 | meta analysis/ | 90,586 |
| 29 | meta-analys$.mp. | 144,878 |
| 30 | exp "systematic review"/ | 47,482 |
| 31 | systematic review$.mp. | 104,640 |
| 32 | exp Guideline/ or exp Practice Guideline/ | 279,209 |
| 33 | guideline$.ti. | 89,070 |
| 34 | random allocation/ | 145,925 |
| 35 | double blind method/ | 302,697 |
| 36 | single-blind method/ | 40,754 |
| 37 | ((singl* or doubl* or trebl* or tripl*) adj25 (blind* or mask*)).mp. [mp=ti, ab, sh, hw, tn, ot, dm, mf, dv, kw, nm, ps, rs, ui, tx, ct] | 475,704 |
| 38 | placebos/ | 210,207 |
| 39 | placebo*.mp. [mp=ti, ab, sh, hw, tn, ot, dm, mf, dv, kw, nm, ps, rs, ui, tx, ct] | 508,565 |
| 40 | random*.mp. [mp=ti, ab, sh, hw, tn, ot, dm, mf, dv, kw, nm, ps, rs, ui, tx, ct] | 1,800,775 |
| 41 | (clinical trial or clinical trials).mp. | 1,700,419 |
| 42 | or/24-41 | 6,745,837 |
| 43 | exp Cohort Studies/ | 1,344,840 |
| 44 | exp longitudinal study/ | 886,305 |
| 45 | exp retrospective study/ | 645,351 |
| 46 | exp prospective study/ | 544,111 |
| 47 | exp observational study/ | 26,520 |
| 48 | exp comparative study/ | 2,211,597 |
| 49 | exp experimental study/ or exp panel study/ or exp quasi experimental study/ | 10,023 |
| 50 | ((clinical or experimental or panel or comparative or cohort or longitudinal or retrospective or prospective or observational or "case-control*" or "cross-over") adj (study or studies or survey or surveys or analysis or analyses or trial or trials)).mp. | 6,249,535 |
| 51 | exp case-control studies/ | 604,497 |
| 52 | exp cross-over studies/ | 83,143 |
| 53 | or/43-52 | 6,612,120 |
| 54 | 23 and (42 or 53) | 1,870 |
| 55 | from 23 keep 1398-2489 | 1,092 |
| 56 | limit 55 to (clinical trial or clinical trial, phase i or clinical trial, phase ii or clinical trial, phase iii or clinical trial, phase iv or comparative study or controlled clinical trial or guideline or meta analysis or multicenter study or practice guideline or randomized controlled trial) (Limit not valid in Embase,CDSR; records were retained) | 480 |
| 57 | 54 or 56 | 1,870 |
| 58 | limit 57 to (editorial or erratum or letter or note or addresses or autobiography or bibliography or biography or comment or dictionary or directory or duplicate publication or interactive tutorial or interview or lectures or legislation or news or newspaper article or overall or patient education handout or periodical index or portraits or published erratum or video-audio media or webcasts) (Limit not valid in Embase,Ovid MEDLINE(R),Ovid MEDLINE(R) In-Process,CCTR,CDSR; records were retained) | 59 |
| 59 | 57 not 58 | 1,811 |
| 60 | from 23 keep 2490-2801 | 312 |
| 61 | 59 or 60 | 1,849 |
| 62 | limit 61 to human (Limit not valid in CCTR,CDSR; records were retained) | 1,634 |
| 63 | limit 62 to yr="2007 -Current" | 622 |
| 64 | limit 63 to humans (Limit not valid in CCTR,CDSR; records were retained) | 622 |
| 65 | remove duplicates from 64 | 426 |

Scopus

| 1 | TITLE-ABS-KEY((influenza* W/2 vaccin*) or (flu W/2 vaccin*) or (flue W/2 vaccin*) or (grippe W/2 vaccin*) or (bronchitis W/1 epidemic* W/2 vaccin*) or (influenza* W/2 immuni*) or (flu W/2 immuni*) or (flue W/2 immuni*) or (grippe W/2 immuni*) or (bronchitis W/1 epidemic* W/2 immuni*) or (influenza* W/2 innoculat*) or (flu W/2 innoculat*) or (flue W/2 innoculat*) or (grippe W/2 innoculat*) or (bronchitis W/1 epidemic* W/2 innoculat*)) |
| --- | --- |
| 2 | TITLE-ABS-KEY(live or attenuated or active) |
| 3 | TITLE-ABS-KEY(killed or inactiv*) |
| 4 | TITLE-ABS-KEY(placebo or placebos) |
| 5 | 2 and (3 or 4) |
| 6 | 3 and 4 |
| 7 | 5 or 6 |
| 8 | 1 and 7 |
| 9 | TITLE-ABS-KEY( (evidence W/1 based) OR (meta W/1 analys*) OR (systematic* W/2 review*) OR guideline OR (control* W/2 stud*) OR (control* W/2 trial*) OR (randomized W/2 stud*) OR (randomized W/2 trial*) or (singl* W/25 blind*) or (doubl* W/25 blind*) or (trebl* W/25 blind*) or (tripl*W/25 blind*) or (singl* W/25 mask*) or (doubl* W/25 mask*) or (trebl* W/25 mask*) or (tripl*W/25 mask*) or random* or placebo* or "clinical trial*") |
| 10 | 8 and 9 |
| 11 | TITLE-ABS-KEY("comparative study" OR "comparative survey" OR "comparative analysis" OR "cohort study" OR "cohort survey" OR "cohort analysis" OR "longitudinal study" OR "longitudinal survey" OR "longitudinal analysis" OR "retrospective study" OR "retrospective survey" or "retrospective analysis" OR "prospective study" OR "prospective survey" OR "prospective analysis" OR "observational study" OR "observational survey" OR "observational analysis" OR "experimental study" OR "experimental analysis" or "panel study" OR "panel survey" OR "panel analysis" or "case-control study" OR "case-control analysis" or "case-controlled study" OR "case-controlled analysis" or "cross-over study" OR "cross-over analysis") |
| 12 | 8 and 11 |
| 13 | 10 or 12 |
| 14 | PMID(0*) OR PMID(1*) OR PMID(2*) OR PMID(3*) OR PMID(4*) OR PMID(5*) OR PMID(6*) OR PMID(7*) OR PMID(8*) OR PMID(9*) |
| 15 | 13 and not 14 |
| 16 | DOCTYPE(le) OR DOCTYPE(ed) OR DOCTYPE(bk) OR DOCTYPE(er) OR DOCTYPE(no) OR DOCTYPE(sh) |
| 17 | 15 and not 16 |
| 18 | PUBYEAR AFT 2006 |
| 19 | 17 and 18 |
